# Differential cytokine expression in gastric tissues highlights *helicobacter pylori*’s role in gastritis

**DOI:** 10.1038/s41598-024-58407-x

**Published:** 2024-04-01

**Authors:** Xing-Tang Yang, Pei-Qin Niu, Xiao-Feng Li, Ming-Ming Sun, Wei Wei, Yan-Qing Chen, Jia-Yi Zheng

**Affiliations:** 1 Department of Gastroenterology, Chongming Branch, Shanghai Tenth People’s Hospital, Tongji University School of Medicine, No. 66 Xiangyangdong Road, Bao Town, Chongming District, Shanghai, 202157 People’s Republic of China; 2 Department of Emergency, Shanghai Tenth People’s Hospital, Tongji University School of Medicine, Shanghai, 200072 People’s Republic of China; 3 Department of Gastroenterology, Shanghai Tenth People’s Hospital, Tongji University School of Medicine, Shanghai, 200072 People’s Republic of China; 4 Department of Pathology, Shanghai Tenth People’s Hospital, Tongji University School of Medicine, Shanghai, 200072 People’s Republic of China

**Keywords:** Gastritis, *Helicobacter pylori*, Cytokines, RT-qPCR, Immunohistochemical staining, H&E staining, Inflammation, ELISA, Gastrointestinal diseases, Bacteria

## Abstract

*Helicobacter pylori* (*H. pylori*), known for causing gastric inflammation, gastritis and gastric cancer, prompted our study to investigate the differential expression of cytokines in gastric tissues, which is crucial for understanding *H. pylori* infection and its potential progression to gastric cancer. Focusing on Il-1β, IL-6, IL-8, IL-12, IL-18, and TNF-α, we analysed gene and protein levels to differentiate between *H. pylori*-infected and non-infected gastritis. We utilised real-time quantitative polymerase chain reaction (RT-qPCR) for gene quantification, immunohistochemical staining, and ELISA for protein measurement. Gastric samples from patients with gastritis were divided into three groups: (1) non-gastritis (N-group) group, (2) gastritis without *H. pylori* infection (G-group), and (3) gastritis with *H. pylori* infection (GH-group), each consisting of 8 samples. Our findings revealed a statistically significant variation in cytokine expression. Generally, cytokine levels were higher in gastritis, but in *H. pylori*-infected gastritis, IL-1β, IL-6, and IL-8 levels were lower compared to *H. pylori*-independent gastritis, while IL-12, IL-18, and TNF-α levels were higher. This distinct cytokine expression pattern in *H. pylori*-infected gastritis underscores a unique inflammatory response, providing deeper insights into its pathogenesis.

## Introduction

Gastric mucosal injury primarily manifests in two forms: gastropathy, which occurs without significant inflammation, and gastritis, which is strongly associated with inflammation^1–3^. Gastritis is commonly attributed to either *Helicobacter pylori* infection or immune-related factors. *H. pylori* is a well-known etiological agent for gastritis, causing either acute or chronic forms of the condition^4^. Acute gastritis presents with distinct symptoms, typically of short duration. With prompt treatment, symptoms alleviate swiftly, enabling a return to normalcy. However, chronic gastritis exhibits a prolonged course, often devoid of acute symptoms initially. Instead, persistent gastric discomfort may ensue over time. Regrettably, chronic gastritis poses challenges in treatment and tends to recur. Helicobacter pylori infection commonly underpins chronic gastritis^5–7^. Chronic gastritis often leads to prolonged symptoms, with patients experiencing discomfort that is reminiscent of functional dyspepsia and, in some cases, symptoms that may be similar to those observed in the early stages of gastric cancer.

*H. pylori* is a significant bacterium colonising the stomachs of approximately 43.1% of the global population^8,9^ and 30.7% in China^9^. Acute gastritis is often characterised by hypochlorhydria and neutrophilic infiltration, which may spontaneously resolve without medical intervention^4^. However, when acute gastritis is not addressed, it tends to progress into chronic active gastritis. This persistent inflammation in chronic active gastritis is a known precursor to the development of gastric cancer^4,10–14^. Neutrophils play a pivotal role as the first line of defence against *H. pylori*-induced gastritis, initiating inflammatory responses crucial in combating the infection^15–18^. Previous studies, including ours, have confirmed the infiltration of CD177 + neutrophils in the gastric mucosa of those affected by *H. pylori*-induced gastritis. This infiltration is a significant factor in the inflammatory process^19,20^. The progression of gastritis to gastric cancer is closely linked to the differential expression of various inflammatory cytokines^20–22^. Key cytokines identified as playing a crucial role in the development and progression of gastritis include IL-1β, IL-6, IL-8, IL-12, IL-18, and TNF-α^23–26^. The roles of these cytokines range from modulating the immune response to directly influencing the inflammatory pathways within the gastric mucosa. Understanding the intricate dynamics of these cytokines in the context of *H. pylori* infection and gastritis can provide valuable insights into the mechanisms underlying the progression to more severe gastric conditions, including gastric cancer.

The research gap in the current literature on gastritis and gastric cancer, particularly those associated with *H. pylori* infection, lies in the limited measurement of cytokine levels in serum rather than in gastric tissues. This gap overlooks the direct environment where these cytokines are most active and influential in the disease process. This study addresses this gap by comparing cytokine levels in the gastric tissues of patients with *H. pylori*-associated gastritis to those with *H. pylori*-independent gastritis. This comparison is crucial for understanding the distinct inflammatory pathways involved in these two types of gastritis and their potential progression to gastric cancer. A comprehensive methodology is employed to achieve this am. Cytokine-related gene expression is quantified using real-time quantitative polymerase chain reaction (RT-qPCR). Additionally, the presence and concentration of cytokine proteins in gastric tissues are assessed qualitatively and quantitatively through immunohistochemical staining and Enzyme-linked immunosorbent assay (ELISA) techniques. This approach not only determines the differences in cytokine levels between *H. pylori*-associated and independent gastritis but also provides insights into the underlying mechanisms driving these conditions.

## Materials and methods

### Gastric tissues

In this study, patients (who visited the gastroenterology department of Shanghai Tenth People’s Hospital, Shanghai, China, from January 2022 to August 2022) experiencing persistent indigestion symptoms such as epigastric discomfort, bloating, and belching underwent an endoscopic examination to diagnose gastritis. Those who didn't respond to standard medication treatments were further evaluated through a biopsy of their gastric tissues. The biopsy samples were used to confirm the presence of gastritis and check for *H. pylori* under a microscope.

Over 8 months at the gastroenterology department of Shanghai Tenth People’s Hospital, we meticulously selected 24 male patients aged 26–79 years for our study, focusing on a homogeneous group to enhance the reliability of our findings and minimise confounding factors. This selection was based on strict inclusion criteria: confirmed *H. pylori* infection through endoscopic and pathological analysis of the gastric mucosa, no usage of proton pump inhibitors, H2 receptor antagonists, antibiotics, or bismuth in the preceding 4 weeks, and willingness to undergo a gastric mucosal biopsy for *H. pylori* culture and drug sensitivity testing. Exclusion criteria included severe organ impairments, complications from peptic ulcers, or mental illnesses affecting accurate symptom reporting. Patients were randomly selected from a pool of 350 eligible individuals using a computer-generated random numbers table to avoid selection bias, aiming to detect significant differences in cytokine expression levels with a predetermined sample size based on preliminary data and power analysis.

For the gastroscopy procedure, subjects were instructed to fast for 12 h beforehand to minimise potential medication interference with the gastric flora. During the procedure, two samples of gastric tissues were collected from each participant from the gastric antrum, approximately 2–3 cm away from the pylorus, ensuring a minimum size of 2 mm × 1 mm. One sample was immediately submerged in liquid nitrogen for gene expression and ELISA studies, while the other was stored in 4% paraformaldehyde for histology and immunohistochemical studies. All samples were transported to the laboratory under cold conditions using dry-ice packs, with samples for gene expression and ELISA stored at − 80 °C and those for histology and IHC studies at 4 °C.

The gastric tissues were divided into three groups: (1) normal tissues as the N-group, (2) *H. pylori*-negative gastritis tissues as the G-group, and (3) *H. pylori*-positive gastritis tissues as the GH-group. This classification aligned with the 'Consensus Opinion for Chronic Gastritis in China, 2012’ by the Chinese Medical Association Gastroenterology Section, ensuring that our study adhered to recognised diagnostic and categorisation criteria. This structured approach allowed for a focused investigation into gastritis’s differential cytokine expression profiles, exploring the immunological mechanisms underlying the clinical presentations of gastritis with and without *H. pylori* infection.

### Histology studies

The histology (using Hematoxylin–eosin staining) of representative tissue sections from each group was shown in the supplementary information, Figure S1. The histological grading of tissue samples (from each group) based on microscopic observation and operative link on gastritis assessment (OLGA) and operative link on gastric intestinal metaplasia (OLGIM) criteria was shown in the supplementary information, Table S1. The endoscopic observations in different parts of gastrointestinal anatomy are shown in the supplementary information, Figure S2 and Table S2. The study was conducted with full consent from all participants and received approval from the Ethics Committee of Shanghai Tenth People’s Hospital (Approval number: SHSY-IEC-4.0/17-101/01), complying with the ethical standards established by China's Human Body Testing Committee.

### Gene expression studies

The expression of cytokines in gastric tissues was quantified using RT-qPCR following the protocols published in the literature^27^. The gastric mucosa tissues were homogenised in liquid nitrogen, and total RNA was extracted (n = 8/group) with Invitrogen TRIzol reagent (ThermoFisher Scientific (China) Co., Ltd, Shanghai, China) according to the manufacturer’s protocol. The concentration and purity of RNA were measured using an Ultra-Micro UV Visible Spectrophotometer (TECAN Infinite M200 Pro). RNA samples (100 ng) were dissolved in 25 µl DNase/RNase-free water and stored at -80˚C until further experimentation. The High Capacity cDNA Reverse Transcription Kit (4374966, ThermoFisher Scientific (China) Co., Ltd, Shanghai, China) was used to synthesise first‑strand cDNAs at 25 ˚C for 5 min, 37 ˚C for 120 min, and 85 ˚C for 5 min in sequence. The qPCR primers (Table 1.) used in the present study are obtained from Shanghai Shenggong Co., Ltd.

**Table 1:** Primer sequences for the qPCR analysis.

Cytokine	Primer	Accession	Base pair
IL-1β^28^	Forward: 5’-TCCCCAGCCCTTTTGTTGA-3’ Reverse: 5’-TTAGAACCAAATGTGGCCGTG-3’	NM_000576.3	91 bp
IL-6^29^	Forward: 5’-TTGTCAAGACATGCCAAAGTG-3’ Reverse: 5’-TCAGACATCTCCAGTCCTATA-3’	AF372214.2	300 bp
IL-8^28^	Forward: 5’-CCAGGAAGAAACCACCGGA-3’ Reverse: 5’-GAAATCAGGAAGGCTGCCAAG-3’	MN930449.1	91 bp
IL-12^30^	Forward: 5’-AGGAATGTTCCCATGCCTTCA-3’ Reverse: 5’-CCAATGGTAAACAGGCCTCCAC-3’	NM_000882.4	170 bp
IL-18^31^	Forward: 5’-TTCCAGATCGCTTCCTCTCGC-3’ Reverse: 5’- GGCCGATTTCCTTGGTCAATG-3’	NM_001386420.1	240 bp
TNF-α^32^	Forward: 5’-TCCCCAGGGACCTCTCTCTA-3’ Reverse: 5’-GAGGGTTTGCTACAACATGGG-3’	NM_000594.4	102 bp
β-actin^31^	Forward: 5’-AAGGCCAACCGCGAGAAGATG-3’ Reverse: 5’-CAGAGGCGTACAGGGATAGCAC-3’	NM_001101	100 bp

qPCR was performed using SYBR® GreenER™ qPCR SuperMix Universal (11762–500, ThermoFisher Scientific (China) Co., Ltd, Shanghai, China) and an Applied CFX96 Real-Time PCR Detection System (Bio-Rad Laboratories, Inc. California, USA). The PCR cycling conditions were 95 ˚C for 60 s, followed by 40 cycles of denaturation at 95 ˚C for 30 s, annealing at 60 ˚C for 25 s, and extension at 72 ˚C for 45 s. The target gene expression was normalised using the reference gene (β-actin). The relative expression of genes was quantified using the E^−ΔΔCt^ method^33,34^. Expression levels of target genes were related to the averaged expression levels of two reference genes^35^. The relative expression of target genes in *Helicobacter pylori*-infected gastritis (GH-group) and normal gastritis (G-group) was compared with those in the normal group (N-group).

### Immunohistochemical staining

The IHC protocol reported in the literature^36,37^ was followed in this study. Freshly dissected gastric tissue (< 3 mm thick) was fixed with 2% paraformaldehyde for 8 h at room temperature. The tissue was rinsed under running tap water for 5 min. Then, the tissue was dehydrated with 70, 80, and 95% ethanol sequentially (5 min each), followed by 100% ethanol (3 times, 5 min each). The tissue was cleared with xylene 2 times (5 min each), followed by immersion in paraffin (3 times, 5 min each) to prepare the paraffin-embedded tissue block. The block was cut into slices (4 μm thickness) using a microtome (Leica RM2235, Leica Biosystems Nussloch GmbH, Germany) and allowed to float on water at 40 °C. The tissue sections were transferred onto IHC microscope slides (FLEX, Agilent) and allowed to dry for 8 h at room temperature. The slides were deparaffinised with xylene (2 times, 5 min each), followed by washing the slides with 100% ethanol (2 times, 3 min each), and then transferred once through 95%, 70% and 50% ethanol (3 min each), respectively. The tissue sections were incubated in H_2_O_2_ solution (3% in methanol) at room temperature for 10 min to block the endogenous peroxidase activity. The slides were then rinsed with phosphate buffer saline (PBS, 2 times, 5 min each). The slides were placed in staining dishes (Thermo Fisher Scientific, USA) and incubated with citrate buffer (10 mM, pH 6.0) at 95 °C for 10 min, followed by removing the slides from the staining dishes and allowing them to cool down to room temperature for about 20 min. The slides were washed with PBS (2 times, 5 min each) followed by incubating them with blocking buffer (100 μL, 10% foetal bovine serum in PBS) at 25 °C for 1 h. The blocking buffer was drained off the slides and incubated with the primary antibody (Abcam (IL-1β, ab9722; IL-6, ab6672; IL-8, ab106350; IL-12, ab9992; IL-18, ab191152; and TNF-α, ab6671), 100 μL, diluted with antibody dilution buffer, 0.5% bovine serum albumin in PBS) at 25 °C for 1 h. The slides were washed with PBS (2 times, 5 min each) and then incubated with biotinylated secondary antibody (Abcam (ab64256),100 μL, diluted with antibody dilution buffer) at 25 °C for 30 min. The slides were washed with PBS (2 times, 5 min each) and incubated in the dark (protecting from light) with Streptavidin–Horseradish Peroxidase (HRP) conjugates (Abcam (ab171537), 100 μL, diluted with antibody dilution buffer) at 25 °C for 30 min. The slides were washed with PBS (2 times, 5 min each) and added 2,4′-dihydroxyacetophenone dioxygenase (DAB) substrate kit (Abcam (ab64238),100 μL, freshly prepared) to allow the colour development for about 5 min. The slides were washed with PBS (3 times, 2 min each) and counterstained with Haematoxylin for 2 min. The slides were then washed under running tap water for 10 min, and the slides were dehydrated using 95% and 100% ethanol (2 times, 5 min each). The tissue slides were cleared with xylene (3 times) and were mounted with a coverslip using a mounting medium (Abcam (ab64320), USA). The slides were observed under the microscope (Nikon Eclipse Ts2-FL, Nikon Instruments Inc. New York, USA)), and the colour intensity was quantified (6 images from random areas of interest at 40X from each tissue) using the software ImageJ Fiji (version 1.2; WS Rasband, National Institute of Health, Bethesda, MD) following the protocol reported in the literature^38–40^.

### Tissue lysate preparation

The tissue lysates were prepared for ELISA. The tissues were rinsed with ice-cold PBS (0.01 M, pH = 7.4) to remove excess hemolysis blood. The tissue pieces were weighed, and minced into smaller pieces. The minced tissue was rinsed thoroughly using PBS. The tissue (100 mg) was homogenised using 500 µL of 1X Cell Extraction Buffer PTR. The samples were incubated on ice for 20 min and centrifuged at 18,000 × g for 20 min at 4 °C. The supernatants were transferred into clean tubes, and the pellets were discarded. The samples were assayed immediately. The protein concentration in the samples was determined using Bradford’s assay following the manufacturer’s instructions^41,42^.

### Protein expression

The inflammatory cytokine proteins (IL-1β, ab214025; IL-6, ab178013; IL-8, ab214030; IL-12, ab223592; IL-18, ab215539; and TNF-α, ab181421) from the tissue lysates were quantified using commercially available enzyme-linked immunosorbent assay kits (ELISA, Abcam, Cambridge, UK) (39–42). Tissue lysates were diluted at least five-fold with 1X Sample Diluent Buffer. Next, in the pre-coated ELISA kit, the standard antibody cocktail (100 μL) and sample solutions (100 μL) were added to the appropriate well (in duplicate), and incubated for 2.5 h at room temperature on a plate shaker with the rotation set at 400 rpm. The contents in the well were washed with wash buffer, and then 100 µL biotinylated detection antibody was added and incubated for 1 h on a shaker set to 400 rpm. The plate was washed with wash buffer, and 100 µL of HRP-streptavidin solution was added and incubated for 45 min at room temperature in the dark. Next, the development solution was added (100 µL) and incubated for 30 min in the dark with shaking, followed by a stop solution (50 µL). The optical density was measured immediately at 450/570 nm using a Spectramax microplate reader M3. The levels of inflammatory cytokines (in pg/mL) in test samples were calculated from the standard curve. The below formula was used to calculate the fold change, and the results are presented as mean ± SD (n = 8/group).

$$Fold change=\frac{\mathrm{ cytokine level in the gastritis group}}{\mathrm{cytokine level normal group}}$$

### Statistical analysi*s*

The statistical analysis used GraphPad Prism version 9.0.1 for Windows (GraphPad Software, San Diego, California, USA). Results are expressed as mean ± standard deviation (SD, n = 8/group). One-way ANOVA followed by Dunnett’s multiple comparisons test was done to determine the statistical significance (P < 0.05).

### Ethics approval and consent to participate

The study protocol was reviewed and approved by the Research and Ethics Committee of the Shanghai Tenth People’s Hospital (Ref: SHSY-IEC-4.0/17–101/01). All the methods were performed in accordance with the relevant guidelines and regulations of ethical approval and ‘Declaration of Helsinki’. The informed consent was obtained from the participants. This study does not involve animals. This study does not contain identifiable human images or data.

## Results

### Histological and endoscopic studies

Hematoxylin–eosin-stained images of the gastric mucosa in different patient groups are shown in Fig. S1. The histological grading of gastric mucosa in different groups of patients is shown in Table S1. Endoscopic features of different parts of gastrointestinal anatomy are shown in Fig. S2, and a comparison of endoscopic observations in different patient groups is in Table S2.

### Differential expression of genes associated with cytokines

**Figure 1:**
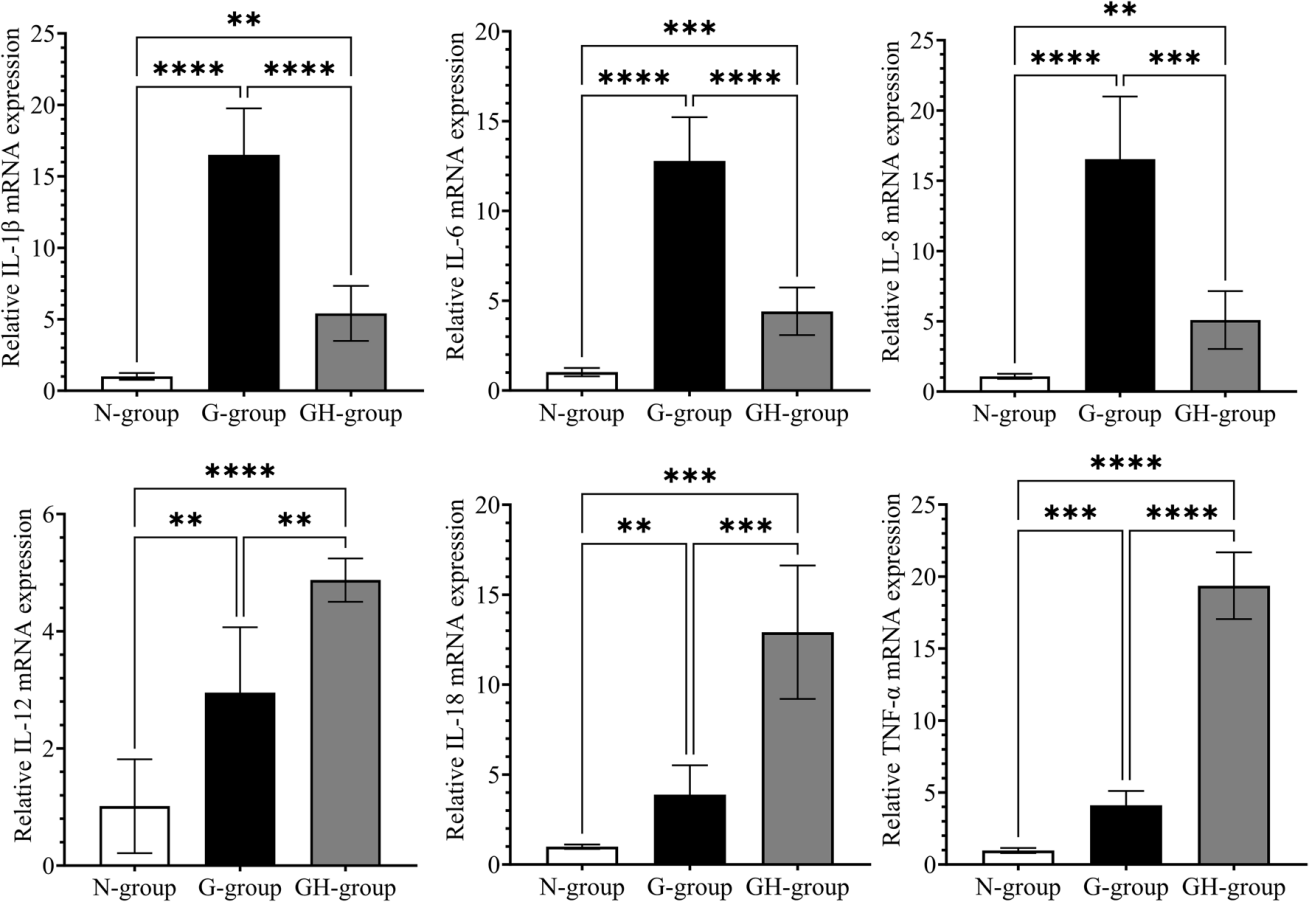
Expression of genes for cytokines in human gastric tissues. The mRNA expression of Interleukin (IL)-1β, IL-6, IL-8, IL-12, IL-18 and tumour necrosis factor (TNF)-α in the normal group (N-group), gastritis without *H. pylori* infection group (G-group) and gastritis with *H. pylori* infection group (GH-group). Target gene expression was calculated with reference to the expression levels of β-actin. A histogram presents data with an error bar (mean ± SD, n = 8/group, *P < 0.05, ** P < 0.01, ***P < 0.001, **** P < 0.0001).

The differential expression of genes for cytokines (IL-1β, IL-6, IL-8, IL-12, IL-18, and TNF-α) in gastric tissues (normal, gastritis, and gastritis with *H. pylori* infection) is shown in Fig. 1.

As presented in Fig. 1, the cytokines (IL-1β, IL-6, IL-8, IL-12, IL-18 and TNF-α) gene expression analysis was performed using RT-qPCR and quantified using the E^-ΔΔCt^ method. Regardless of the presence of *H. pylori* infection, it was observed that the cytokines gene levels in gastritis were upregulated, and the differences are statistically significant (P < 0.001). Further analysis in which *H. pylori* infection was present showed statistically significant (P < 0.001) differences in the comparison between non-*H. pylori* and *H. pylori*-infected gastritis. The IL-1β, IL-6, and IL-8 levels were lower in *H. pylori*-infected gastritis compared to non-infected gastritis. In contrast, the IL-12, IL-18, and TNF-α levels were higher in *H. pylori*-infected gastritis compared to non-infected gastritis.

### Immunohistochemical analysis of inflammatory cytokines expression

**Figure 2:**
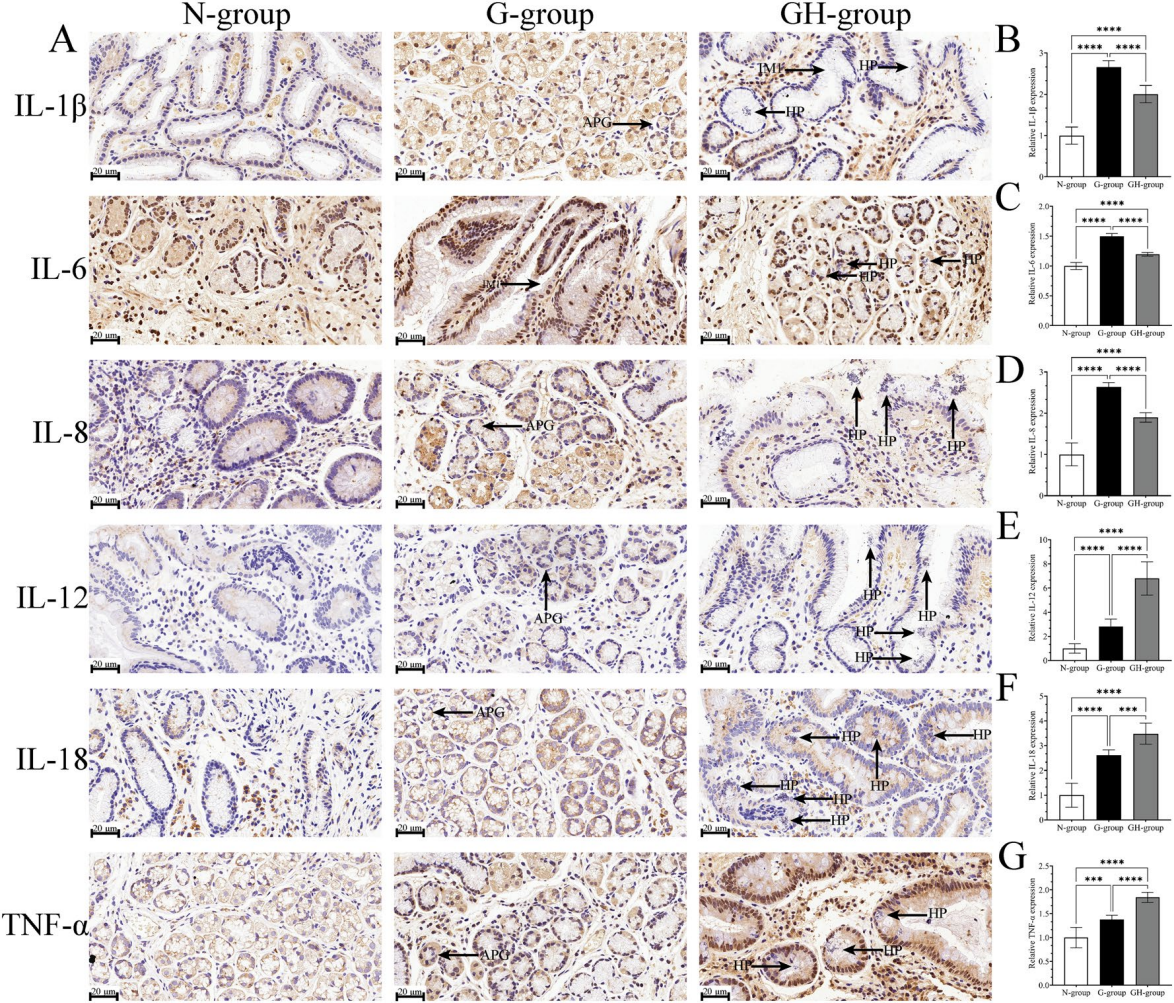
Immunohistochemistry of the gastric mucosa. (**A**) Representative IHC images Interleukin (IL) -1β, IL-6, IL-8, IL-12, IL-18, and Tumor necrosis factor-α (TNF-α). (**B**-**G**) Quantitative analysis of IL-1β, IL-6, IL-8, IL-12, IL-18, and TNF-α expression in gastric mucosa. The data are expressed as the means ± SD (n = 8/group). *p < 0.05, **p < 0.01, ***p < 0.001, ****p < 0.0001. N-group, normal group; G-group, gastritis without *H. pylori* infection; GH-group, gastritis with *H. pylori* infection. IM1: intestinal metaplasia; APG: atrophy of pyloric gland; HP: *Helicobacter pylori*.

As presented in Fig. 2, the histochemical staining technique was performed for the qualitative and semi-quantitative analysis of cytokines (1β, IL-6, IL-8, IL-12, IL-18 and TNF-α) expression. The protein expression trend was consistent with that observed in gene expression studies. The cytokines were generally expressed higher in gastritis compared to normal gastric tissues. IL-1β, IL-6, and IL-8 were lower in *H. pylori* infection-associated gastritis samples, whereas the levels of IL-12, IL-18, and TNF-α were higher.

### Quantification of proteins (ELISA)

**Figure 3:**
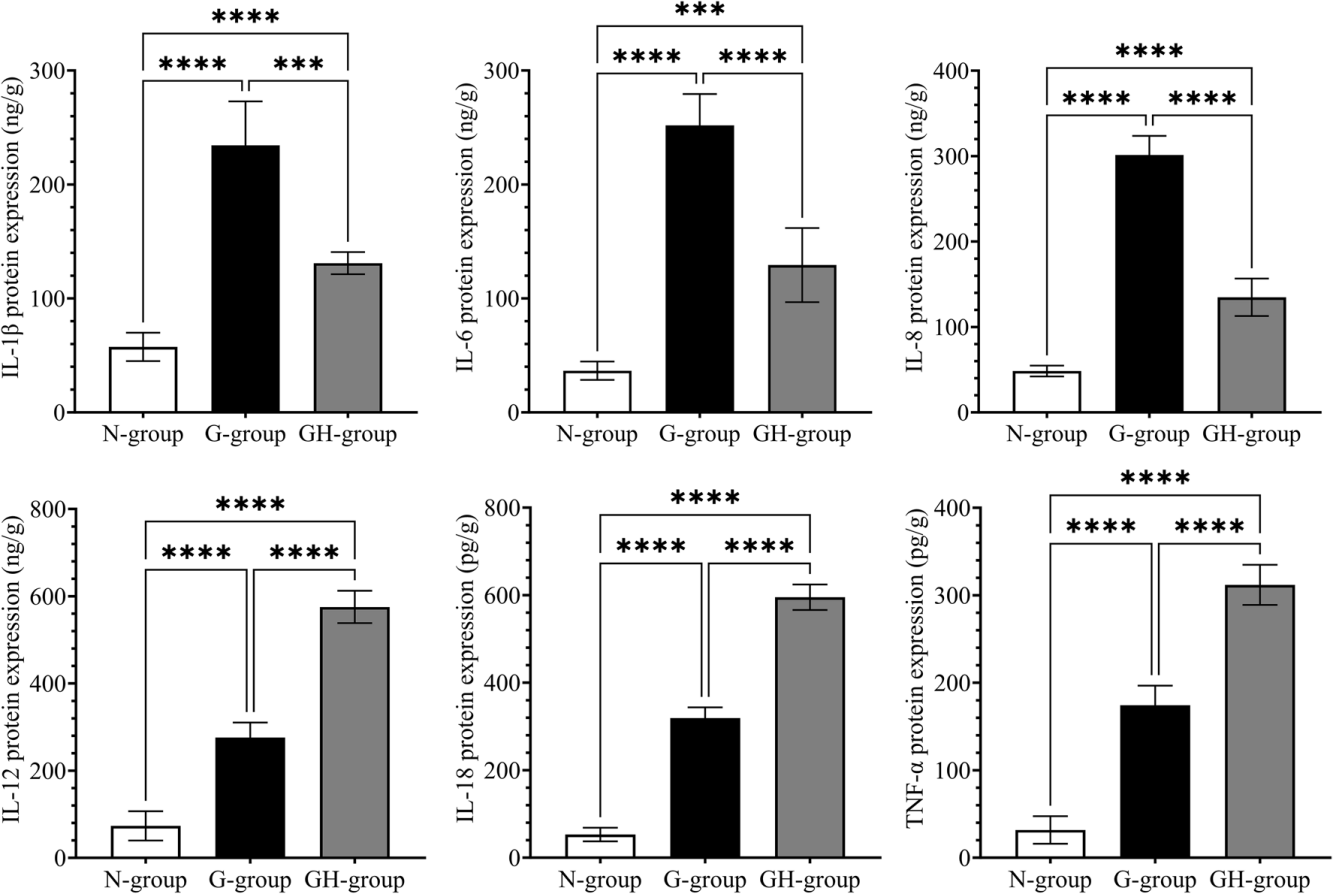
Quantitative analysis (ELISA) of Interleukin-1β (IL-1β), IL-6, IL-8, IL-12, IL-18 and tumor necrosis factor (TNF)-α. The expression of proteins was normalised to unit protein concentration. The histogram presents data with an error bar. (Mean ± SD, n = 8/group, *P < 0.05, ** P < 0.01, ***P < 0.001, **** P < 0.0001). N-group, normal group; G-group, gastritis without *H. pylori* infection; GH-group, gastritis with *H. pylori* infection.

Commercially available ELISA plates were used to confirm further the influence of *H. pylori* infection on the expression of cytokines (IL-1β, IL-6, IL-8, IL-12, IL-18, and TNF-α) in gastritis. As presented in Fig. 3. The results agree with those observed in immunohistochemical staining. All the cytokines were elevated in the gastritis samples. In *H. pylori* infection-associated gastritis, the IL-1β, IL-6, and IL-8 were lower, whereas IL-12, IL-18, and TNF-α were higher than those in gastritis without *H. pylori* infection.

## Discussion

In our previous paper^43^, we presented the characteristics and clinical pictures of the patients suffering from gastritis (with and without *H. pylori* infection). In gastritis patients without *H. pylori* infection, 67.6% showed grade 1, 20.6% showed grade 2, and 11.8% showed grade 3 inflammation. In *H. pylori*-infected gastritis patients, 24.4% showed grade 1, 46.1% showed grade 2, and 28.9% showed grade 3 inflammation. In light of these findings, our current study extends the investigation to understand how these varying degrees of inflammation correlate with the differential cytokine expression profiles in gastritis. This study allows us to explore the underlying immunological mechanisms that may drive the differences observed in clinical presentations of gastritis with and without *H. pylori* infection.

The intricate interplay of cytokines plays a pivotal role in the pathogenesis of gastritis, a condition characterised by gastric mucosal inflammation. Our investigation delves into the differential expression of key cytokines in gastric tissue, including IL-1β, IL-6, IL-8, IL-12, IL-18, and TNF-α in the context of *H. pylori*-associated and *H. pylori*-independent gastritis. This exploration is crucial, as cytokines are not only markers of inflammation but also potent mediators of the immune response, influencing the disease's trajectory and its potential progression to more severe conditions, including gastric cancer.

The etiology of gastritis is a complex interplay of genetic predisposition and environmental influences. The genetic polymorphisms in proinflammatory cytokines and variations in gastric acid secretion due to genetic factors significantly influence gastritis's onset and progression^44^. The pronounced roles of IL-6 and IL-12 in exacerbating gastric inflammation is evident from the literature^43,45^. IL-6, a key proinflammatory cytokine, has been linked with the severity of inflammation and may serve as a biomarker for the progression of gastritis. Similarly, IL-12, known for its role in Th1 cell differentiation, appears to be a critical player in the inflammatory milieu of gastritis. The pivotal roles of specific cytokines in gastric cancer-related inflammation are reported elsewhere in the literature. IL-1β, typically a macrophage product with antitumor properties, paradoxically promotes tumorigenesis under chronic inflammatory conditions, indicating its dual role in gastric pathophysiology^45,46^.

Similarly, IL-18 and TNF-α, both implicated in tumour growth and angiogenesis, point to a complex network of cytokine interactions driving the progression from chronic inflammation to cancer^45^. The dynamic interplay between cytokines that promote and suppress antitumour activity is critical to our research. Cytokines such as IFN-γ, IL-12, and IL-18 demonstrate antitumour effects, while IL-10 is known to inhibit T-cell synthesis and inflammatory cytokine secretion, suggesting its role in suppressing antitumour activity^45^.

*H. pylori*, a well-established factor in gastric carcinogenesis, is responsible for a significant proportion of gastric malignancies. *H. pylori* initiates a cascade of molecular and morphological changes, leading to gastric cancer^45,47^. The progression of gastritis is influenced by *H. pylori* infection, and cytokine profiles, as observed in a study, follow a distinct histopathological trajectory from chronic active non-atrophic gastritis to gastric cancer^45,48^. The bacterium's ability to alter the gastric environment and affect the resident microflora is a crucial aspect of gastritis pathogenesis, with implications for gastric cancer development^45,49,50^.

In our experimental study, IL-8 levels in gastritis were found to be elevated compared to normal conditions, with a notable observation that IL-8 levels in *H. pylori*-associated gastritis were lower than those in *H. pylori*-independent gastritis. This finding adds complexity to the already established role of IL-8 in gastritis, where it is known to be one of the first cytokines produced by infected gastric epithelium and plays a significant role in the inflammatory response^51^. Gastric epithelial cells act as a major source of IL-8, and its secretion leads to the activation of inflammatory cells within *H. pylori*-infected mucosa. Additionally, IL-8 facilitates the recruitment of leukocytes to the gastric mucosa, a crucial step in regulating immune-inflammatory responses. The signalling pathways of IL-8, predominantly through NF-κB, are significant in its activation. The IL-8 gene polymorphism further influences its activity and the inflammatory response, with certain genetic variations being linked to increased susceptibility to *H. pylori* infection and persistence^52^. Gender differences also appear to play a role in the influence of IL-8 gene polymorphism on gastritis severity^53^. However, the association between IL-8 gene polymorphisms and *H. pylori* infection is debated in the literature^54,55^. Recent studies have highlighted that *H. pylori*-derived outer membrane vesicles increase IL-8 mRNA expression and secretion, indicating a broader impact of *H. pylori* on gastric epithelial cells^56^. On the contrary, a study reported a negative correlation between *H. pylori* nodular gastritis and IL-8 mRNA expression, however, a general increase in IL-8 expression in *H. pylori*-positive patients^57^. This complex interplay of factors challenges the initial perception of IL-8's role in *H. pylori* infection, as evidenced by research showing varying effects of IL-8 based on host immune responses and bacterial characteristics^58^.

Our experimental study observed that IL-8 levels were elevated in gastritis compared to normal conditions. Interestingly, IL-8 levels in *H. pylori*-associated gastritis were lower than in *H. pylori*-independent gastritis. This finding complements the established knowledge that IL-6, a multifunctional cytokine expressed by various cells such as monocytes, lymphocytes, and macrophages, is crucial in immunity and inflammation^59,60^. It significantly stimulates acute-phase reactants, indicating its involvement in inflammatory responses. In the context of *H. pylori* infection, elevated IL-6 levels have been consistently reported, with a notable association between serum IL-6 levels and *H. pylori* antibodies. This observation underscores its importance in the inflammatory process of *H. pylori*-positive gastritis^61^. Moreover, gastric mucosal levels of IL-6 are found to be higher in patients with *H. pylori*-positive gastritis and decrease following successful eradication of the infection^62,63^. These observations align with our findings of altered cytokine levels in different types of gastritis, highlighting the complex interplay of cytokines in the pathogenesis of this condition.

In our experimental study, we observed that IL-1β levels were increased in gastritis compared to normal, yet interestingly, IL-1β levels in *H. pylori*-associated gastritis were lower than in *H. pylori*-independent gastritis. This finding adds nuance to the established role of IL-1β as a significant trigger and enhancer of inflammation produced by various cells, including immune cells, epithelial cells, and fibroblasts^64^. It is known to augment the inflammatory response to stimuli and induce the expression of other cytokines and proinflammatory mediators^65,66^. IL-1β's role in the innate immune system is highlighted by its expression regulated by toll-like receptors 2 and 4 in *H. pylori*-infected pediatric patients^67^. Moreover, its involvement in the pathogenesis of peptic ulcers, irrespective of *H. pylori* infection, aligns with our observations, suggesting a complex relationship between IL-1β levels and *H. pylori* status in gastritis. The IL-1β’s varied expression in different gastritis contexts underscores its pivotal role in the inflammatory processes associated with this condition.

Our experimental study found that IL-18 levels were elevated in gastritis compared to normal, with even higher levels observed in *H. pylori*-associated gastritis than in *H. pylori*-independent gastritis. This observation aligns with the known role of IL-18 in the inflammatory processes of gastritis, as IL-18 produces proangiogenic factors and thrombospondin in gastric cells, affecting angiogenesis through the JNK pathway^59^. Additionally, IL-18's capability to amplify gastric cell migration and activate the NF-κB pathway suggests its significant involvement in the inflammatory response associated with *H. pylori* infection^59^. As observed in our study, the elevated levels of IL-18 in *H. pylori*-associated gastritis underscore its potential role in the severity and progression of gastritis, particularly in the context of *H. pylori* infection.

In our experimental study, we observed an increase in TNF-α levels in gastritis compared to normal, with notably higher levels in *H. pylori*-associated gastritis than in *H. pylori*-independent gastritis. This observation aligns with the established role of TNF-α as a key mediator of systemic inflammation and acute phase reactions in the context of gastritis^68,69^. Specifically, TNF-α, elevated in response to *H. pylori* lipopolysaccharide stimulation of macrophages, plays a crucial role in the inflammatory responses to *H. pylori*^70^. Its recruitment and activation of monocytes, macrophages, and neutrophils and its significantly higher levels in patients with *H. pylori* infection than healthy controls underscores its importance in gastritis. The correlation between TNF-α expression and bacterial load^57^ and its association with the severity of tissue damage in peptic ulcer patients^71^ further support our findings of elevated TNF-α levels in *H. pylori*-associated gastritis, indicating a profound role of TNF-α in the inflammatory processes of this condition.

Our study's investigation into the differential cytokine expression in *H. pylori*-associated and independent gastritis holds significant importance in the current understanding of *H. pylori* pathogenesis and gastritis. This research is a critical contribution to the field, focusing on the complex interactions between *H. pylori* and the host immune system, particularly highlighting the role of cytokines. Our study provides invaluable insights into the inflammatory mechanisms involved by exploring key cytokines such as IL-1β, IL-6, IL-8, IL-12, IL-18, and TNF-α in gastritis. These insights are crucial as cytokines are not only markers of inflammation but also active mediators of the immune response, influencing the progression of gastritis and potentially leading to more severe conditions like gastric cancer. Our study's approach to analysing cytokine expression concerning the severity of gastritis, identified through graded inflammation levels, offers a more comprehensive and nuanced understanding of the disease's pathogenesis.

Our study's limitation lies in not fully evaluating the spectrum of factors influencing the pathogenicity of *H. pylori* in associated gastritis^45^. The colonisation efficiency of *H. pylori* in the stomach mucosal layer is affected by bacterial motility, influenced by mutations in genes encoding flagellar proteins like fliD, FlaA, and FlaB, which can impair its infection capability^72^. *H. pylori's* chemotactic responses to mucin and sodium bicarbonate molecules are also significant. The cytotoxin-associated gene (cag) pathogenicity island, encoding adhesins like BabA, OipA, and SabA, is essential for adhesion to gastric epithelial cells and has implications in inflammation and cancer development. This island also encodes the type IV secretion system (T4SS) and oncoproteins CagA and VacA, influencing the risk of gastric precancerous lesions and adenocarcinoma^73–75^. The effects of CagA and VacA on epithelial cell morphology, intracellular signalling, and gastric epithelial permeability highlight their roles in *H. pylori* pathogenicity^76–79^. Genetic polymorphisms in cytokines like IL-1β, IL-1 receptor antagonist, TNF-α, and IL-10 are linked to inflammatory response modulation and carcinogenesis susceptibility. At the same time, the potential role of EBV in gastric cancer adds another dimension to gastric carcinogenesis^80^. Higher *H. pylori* infection rates among first-degree relatives of gastric cancer patients suggest a genetic predisposition impacted by dietary and lifestyle factors, such as high salt intake, smoking, and post-surgical bile reflux^81^. The discovery of diverse microbial communities in the gastric juice and mucosa challenges the notion of a sterile stomach and contributes to the risk of gastric cancer^82–85^. Influences on the gastric microbiome, such as age, diet, medication use, and *H. pylori* presence, emphasise the complexity of its role in gastritis and gastric cancer progression^50,86–89^. This intricate interplay of bacterial motility, adhesion, virulence factors, genetic polymorphisms, and environmental and lifestyle factors underscores the multifaceted nature of *H. pylori* pathogenesis in gastritis and its progression to more severe conditions like gastric cancer.

Future research should adopt a more comprehensive approach to address the limitations identified in our study and further elucidate the pathogenicity of *H. pylori* in gastritis, including examining the genetic variations in flagellar proteins like fliD, FlaA, and FlaB that affect *H. pylori* motility, and exploring the bacterium's chemotactic responses to mucin and sodium bicarbonate. Investigating the role of the cytotoxin-associated gene (cag) pathogenicity island is crucial, particularly focusing on adhesins like BabA, OipA, and SabA and their implications in inflammation and cancer development. Additionally, the impact of oncoproteins CagA and VacA on epithelial cells, including morphological changes and intracellular signalling, warrants detailed study. Genetic polymorphisms in cytokines related to inflammatory response and carcinogenesis susceptibility, such as IL-1β, TNF-α, and IL-10, should be a focal point. Investigating the potential genetic predisposition to *H. pylori* infection, particularly in first-degree relatives of gastric cancer patients, along with the influence of dietary and lifestyle factors, will also be essential. Furthermore, exploring the diverse microbial communities in the gastric environment and their influence on the gastric microbiome, considering factors like age, diet, and medication use, will enhance our understanding of gastritis and gastric cancer progression. These multifaceted aspects, if thoroughly researched, could provide significant insights into the complex interplay of factors influencing *H. pylori*-associated gastritis and its potential progression to gastric cancer.

## Conclusion

In this study, we have made significant strides in understanding the immune responses in gastritis, particularly in the context of *H. pylori* infection. We focused on the differential cytokine expression in *H. pylori*-associated and non-associated cases of gastritis. Our observations revealed distinct expression patterns of cytokines such as IL-1β, IL-6, IL-8, IL-12, IL-18, and TNF-α, highlighting notable differences between H. pylori-infected and non-infected groups. These findings contribute to a deeper understanding of the complex immunological mechanisms underpinning gastritis and its potential progression to gastric cancer.

However, our study is not without its limitations. Our work does not fully explore all factors influencing *H. pylori* pathogenicity, including an in-depth investigation into genetic polymorphisms in cytokines and the bacterium's interaction with the gastric microbiome. The intricate interplay of bacterial motility, adhesion, virulence, and the influence of environmental and lifestyle factors creates a multifaceted landscape for *H. pylori* pathogenesis in gastritis.

Given these limitations, we propose several critical areas for future research. First, there is a need for a comprehensive approach to examine the genetic variations affecting *H. pylori* motility and the role of the cag pathogenicity island in inflammation and cancer development. Future studies should also investigate the effects of lifestyle and dietary factors on *H. pylori* infection and the complex interactions within the gastric microbiome. A comparative study of cytokine levels in gastric sections versus sera is also crucial. This approach could provide insights into systemic versus local immune responses in gastritis, uncovering nuanced aspects of the immune response mechanisms. We also advocate for the refinement of diagnostic criteria for *H. pylori* infection. Introducing additional, more precise diagnostic markers would allow for more accurate differentiation between the acute and chronic stages of the infection, facilitating a more tailored approach to treatment.

These future research directions are essential to address the current study's limitations and broaden the understanding of gastritis pathophysiology. Ultimately, this holistic perspective is crucial for the development of more effective diagnostic and therapeutic strategies in the field, advancing our understanding of gastritis and its potential progression to more severe conditions like gastric cancer.

### Supplementary Information

Supplementary Information.

## Data Availability

The datasets used and analysed during the current study are available from the corresponding author upon reasonable request.

## References

[1] Carpenter HA, Talley NJ (1995). Gastroscopy is incomplete without biopsy: Clinical relevance of distinguishing gastropathy from gastritis. Gastroenterology.

[2] Kayaçetin S, Güreşçi S (2014). What is gastritis? What is gastropathy? How is it classified?. Turkish J. Gastroenterol..

[3] Dixon MF (1996). Classification and grading of gastritis: The updated sydney system. Am. J. Surg. Pathol..

[4] Watari J (2014). Helicobacter pylori associated chronic gastritis, clinical syndromes, precancerous lesions, and pathogenesis of gastric cancer development. World J. Gastroenterol..

[5] Eusebi LH (2018). Global prevalence of, and risk factors for, gastro-oesophageal reflux symptoms: A meta-analysis. Gut.

[6] Barberio B, Mahadeva S, Black CJ, Savarino EV, Ford AC (2020). Systematic review with meta-analysis: global prevalence of uninvestigated dyspepsia according to the Rome criteria. Aliment. Pharmacol. Ther..

[7] Ford AC, Marwaha A, Sood R, Moayyedi P (2015). Global prevalence of, and risk factors for, uninvestigated dyspepsia: A meta-analysis. Gut.

[8] Butt J, Epplein M (2023). How do global trends in <em>Helicobacter pylori</em> prevalence inform prevention planning?. Lancet Gastroenterol. Hepatol..

[9] Zou J-C (2023). Helicobacter pylori infection prevalence declined among an urban health check-up population in Chengdu, China: A longitudinal analysis of multiple cross-sectional studies. Front. Public Health.

[10] Yasmin R (2015). Epigenetic regulation of inflammatory cytokines and associated genes in human malignancies. Mediat. Inflamm..

[11] Fernandes JV (2015). The role of the mediators of inflammation in cancer development. Pathol. Oncol. Res..

[12] Wang YK (2022). How does helicobacter pylori infection cause gastric mucosal atrophy. Infect. Drug Resist..

[13] Jaroenlapnopparat A, Bhatia K, Coban S (2022). Inflammation and Gastric Cancer. Diseases.

[14] Nardone G, Rocco A, Malfertheiner P (2004). Review article: *Helicobacter pylori* and molecular events in precancerous gastric lesions. Aliment. Pharmacol. Ther..

[15] Choli-Papadopoulou T, Kottakis F, Papadopoulos G, Pendas S (2011). *Helicobacter pylori* neutrophil activating protein as target for new drugs against H. Pylori inflammation. World J. Gastroenterol..

[16] Moyat M, Velin D (2014). Immune responses to Helicobacter pylori infection. World J. Gastroenterol..

[17] D'Elios MM, Amedei A, Cappon A, Del Prete G, De Bernard M (2007). The neutrophil-activating protein of Helicobacter pylori (HP-NAP) as an immune modulating agent. FEMS Immunol. Med. Microbiol..

[18] Eletto D (2022). *Helicobacter pylori* pathogen-associated molecular patterns: Friends or foes?. Int. J. Mol. Sci..

[19] Yang XT, Wang ZJ (2019). CD177 Expression and inflammation grade in *Helicobacter pylori*-infected wild-type and CD177-/-C57BL/6 Mice. Anal. Cell. Pathol..

[20] Jan I (2021). Helicobacter pylori subdues cytokine signaling to alter mucosal inflammation via hypermethylation of suppressor of cytokine signaling 1 gene during gastric carcinogenesis. Front. Onco..

[21] Bockerstett KA, DiPaolo RJ (2017). Regulation of gastric carcinogenesis by inflammatory cytokines. CMGH.

[22] Sun X (2019). Relationship between serum inflammatory cytokines and lifestyle factors in gastric cancer. Mol. Clin. Oncol..

[23] Esedov EM, Akbieva DS (2019). Proinflammatory cytokines in the gastric juice in acid-related diseases before and after standart therapy. Klin Lab Diagn..

[24] Mosiychuk LM, Tatarchuk OM, Petishko OP (2022). Gender features of the cytokine profile in patients with chronic atrophic gastritis. Gastroenterology.

[25] Yu B, Xiang L, Peppelenbosch MP, Fuhler GM (2023). Overlapping cytokines in H. pylori infection and gastric cancer: A tandem meta-analysis. Front. Immunol..

[26] Davari F, Shokri-Shirvani J, Sepidarkish M, Nouri HA-O (2023). Elevated expression of the AIM2 gene in response to *Helicobacter pylori* along with the decrease of NLRC4 inflammasome is associated with peptic ulcer development. J. Pathol. Microbiol. Immunol..

[27] Vandesompele J (2002). Accurate normalisation of real-time quantitative RT-PCR data by geometric averaging of multiple internal control genes. Genome Biol..

[28] Outlioua A (2020). Gastric IL-1β, IL-8, and IL-17A expression in Moroccan patients infected with Helicobacter pylori may be a predictive signature of severe pathological stages. Cytokine.

[29] Lobo Gatti L (2005). Interleukin-6 polymorphism and Helicobacter pylori infection in Brazilian adult patients with chronic gastritis. Clin. Exp. Med..

[30] Al-Sammak F (2013). Gastric epithelial expression of IL-12 Cytokine family in helicobacter pylori infection in Human: Is it head or tail of the coin?. PLOS ONE.

[31] Kimang’a A (2010). IL-17A and IL-17F gene expression is strongly induced in the mucosa of h pylori-infected subjects from Kenya and Germany. Scandinavian J. Immunol..

[32] Boccellato F (2019). Polarised epithelial monolayers of the gastric mucosa reveal insights into mucosal homeostasis and defence against infection. Gut.

[33] Saviozzi S (2006). Selection of suitable reference genes for accurate normalisation of gene expression profile studies in non-small cell lung cancer. BMC Cancer.

[34] Hellemans J, Mortier G, De Paepe A, Speleman F, Vandesompele J (2008). qBase relative quantification framework and software for management and automated analysis of real-time quantitative PCR data. Genom. Biol..

[35] Hoen T, C PA (2003). Aorta of deficient mice responds to atherogenic stimuli by a prelesional increase and subsequent decrease in the expression of antioxidant enzymes. Circ. Res..

[36] Kim SW, Roh J, Park CS (2016). Immunohistochemistry for pathologists: Protocols, pitfalls, and tips. J. Pathol. Trans. Med..

[37] Zhou CH, Liu LL, Wu YQ, Song Z, Xing SH (2012). Enhanced expression of salusin-β contributes to progression of atherosclerosis in LDL receptor deficient mice. Can. J. Physiol. Pharmacol..

[38] Crowe A, Yue W (2019). Semi-quantitative Determination of Protein Expression Using Immunohistochemistry Staining and Analysis: An Integrated Protocol. Bio-Protocol.

[39] Sharma N (2019). Pharmacological inhibition of Notch signaling regresses pre-established abdominal aortic aneurysm. Sci. Rep..

[40] Sachindelin J (2012). Fiji: An open-source platform for biological-image analysis. Nat. Methods.

[41] Ernst O, Zor T (2010). Linearization of the bradford protein assay. J. Visual. Exp..

[42] Kielkopf CL, Bauer W, Urbatsch IL (2020). Bradford assay for determining protein concentration. Cold Spring Harbor protocol..

[43] Yang X (2017). Increased CD177 expression is associated with helicobacter pylori-related gastritis. Int. J. Clin. Exp. Pathol..

[44] Watari J (2014). Helicobacter pylori associated chronic gastritis, clinical syndromes, precancerous lesions, and pathogenesis of gastric cancer development. World J. Gastroenterol..

[45] Jaroenlapnopparat A, Bhatia K, Coban S (2022). Inflammation and Gastric Cancer. Diseases.

[46] Ma J (2021). Interleukin-1 receptor antagonist inhibits metastatic potential by down-regulating CXCL12/CXCR4 signaling axis in colorectal cancer. Cell Commun. Signal..

[47] Plummer M, Franceschi S, Vignat J, Forman D, de Martel C (2015). Global burden of gastric cancer attributable to Helicobacter pylori. Int. J. Cancer.

[48] Battista S, Ambrosio MR, Limarzi F, Gallo G, Saragoni L (2021). Molecular alterations in gastric preneoplastic lesions and early gastric cancer. Int. J. Mol. Sci..

[49] Mitchell DR (2017). The gastric acid pocket is attenuated in H. pylori infected subjects. Gut.

[50] Li TH (2017). Alterations in gastric microbiota after H. pylori eradication and in different histological stages of gastric carcinogenesis. Sci. Rep..

[51] Dincă AL, Meliț LE, Mărginean CO (2022). Old and new aspects of H. pylori-associated inflammation and gastric cancer. Children.

[52] Ramis IB (2017). Polymorphisms of the IL-6, IL-8 and IL-10 genes and the risk of gastric pathology in patients infected with Helicobacter pylori. J. Microbiol. Immunol. Infect..

[53] Barboza MM, Barbosa FC, do Carmo APS, Barroso FC, Rabenhorst SHB (2021). Contribution of genetic polymorphisms of interleukins IL1B-511 C/T, IL1RN VNTR, IL6-174 G/C, and IL8-251 A/T in gastric lesions: gender and Helicobacter pylori genes matter. Arch. Microbiol..

[54] Xue H (2012). A meta-analysis of interleukin-10 -592 promoter polymorphism associated with gastric cancer risk. PLOS ONE.

[55] Zhao Y (2013). Association between TNF-α and IL-1β genotypes vs Helicobacter pylori infection in Indonesia. World J. Gastroenterol..

[56] Choi MS, Ze EY, Park JY, Shin TS, Kim JG (2021). Helicobacter pylori derived outer membrane vesicles stimulate interleukin secretion through nuclear factor kappa B activation. Korean J. Inter. Med..

[57] Mansilla-Vivar R (2020). High helicobacter pylori bacterial load and low cytokine expression levels are associated with nodular Gastropathy. Dig. Dis. Sci..

[58] Chen S-T, Ni Y-H, Li C-C, Liu S-H (2018). Hepcidin correlates with interleukin-1β and interleukin-6 but not iron deficiency in children with *Helicobacter pylori* infection. Pediatr. Neonatol..

[59] Bagheri V (2018). Cytokine networks and their association with *Helicobacter pylori* infection in gastric carcinoma. J. Cell. Physiol..

[60] Taniguchi K, Karin M (2014). IL-6 and related cytokines as the critical lynchpins between inflammation and cancer. Seminars Immunol..

[61] Nakagawa H (2013). Significant association between serum interleukin-6 and helicobacter pylori antibody levels among H. Pylori-positive Japanese adults. Mediat. Inflam..

[62] Sugimoto M, Yamaoka Y, Furuta T (2010). Influence of interleukin polymorphisms on development of gastric cancer and peptic ulcer. World J. Gastroenterol..

[63] Mejías-Luque R, Peiró S, Vincent A, Van Seuningen I, de Bolós C (2008). IL-6 induces MUC4 expression through gp130/STAT3 pathway in gastric cancer cell lines. Biochimica et Biophysica Acta (BBA) Mol. Cell Res..

[64] Arango Duque G, Descoteaux A (2014). Macrophage cytokines: Involvement in immunity and infectious diseases. Front. Immunol..

[65] Garlanda C, Dinarello CA, Mantovani A (2013). The Interleukin-1 Family: Back to the future. Immunity.

[66] Turner MD, Nedjai B, Hurst T, Pennington DJ (2014). Cytokines and chemokines: At the crossroads of cell signalling and inflammatory disease. Biochim. Biophys. Acta (BBA) Mol. Cell Res..

[67] Hong J-B, Zuo W, Wang A-J, Lu N-H (2016). *helicobacter pylori* infection synergistic with il-1β gene polymorphisms potentially contributes to the carcinogenesis of gastric cancer. Int. J. Med. Sci..

[68] Fond G (2014). Effectiveness and tolerance of anti-inflammatory drugs' add-on therapy in major mental disorders: A systematic qualitative review. Acta Psychiatrica Scandinavica.

[69] Güzel M (2016). Effectiveness of lycopene on experimental testicular torsion. J. Pediatric. Surg..

[70] Moradipour A, Khosravi A, Piri F (2018). Fecal Helicobacter pylori glmM and 16S rRNA genes correlate with serum TNF-α and IL-1β cytokine fluctuations. Acta Microbiologica et Immunologica Hungarica.

[71] Tourani M (2018). Association of TNF but not IL levels with the presence of Helicobacter pylori infection increased the risk of peptic ulcer development. Cytokine.

[72] Gu H (2017). Role of flagella in the pathogenesis of helicobacter pylori. Curr. Microbiol..

[73] Vinella D (2015). Evolution of helicobacter: Acquisition by gastric species of two histidine-rich proteins essential for colonization. PLOS Pathogens.

[74] Ferreira RM, Machado JC, Figueiredo C (2014). Clinical relevance of *Helicobacter pylori* vacA and cagA genotypes in gastric carcinoma. Best Pract. Res. Clin. Gastroenterol..

[75] González CA (2011). Helicobacter pylori caga and vaca genotypes as predictors of progression of gastric preneoplastic lesions: A long-term follow-up in a high-risk area in Spain. Off. J. Am. Coll. Gastroenterol..

[76] Chang C-C (2016). Fragmentation of CagA Reduces Hummingbird Phenotype Induction by *Helicobactor pylori*. PLOS ONE.

[77] Tegtmeyer N, Wessler S, Backert S (2011). Role of the cag-pathogenicity island encoded type IV secretion system in *Helicobacter pylori* pathogenesis. FEBS J..

[78] Queiroz DMM (2012). Higher frequency of cagA EPIYA-C Phosphorylation Sites in H. pylori strains from first-degree relatives of gastric cancer patients. BMC Gastroenterol..

[79] Coulombe G (2016). SHP-2 phosphatase prevents colonic inflammation by controlling secretory cell differentiation and maintaining host-microbiota homeostasis. J. Cell. Physiol..

[80] Shinozaki-Ushiku A, Kunita A, Fukayama M (2015). Update on Epstein-Barr virus and gastric cancer (Review). Int. J. Oncol..

[81] Hwang TL (2012). CCL7 and CCL21 overexpression in gastric cancer is associated with lymph node metastasis and poor prognosis. World J. Gastroenterol..

[82] Delgado S, Cabrera-Rubio R, Mira A, Suárez A, Mayo B (2013). Microbiological survey of the human gastric ecosystem using culturing and Pyrosequencing methods. Microb. Ecol..

[83] Sung J (2016). Comparison of gastric microbiota between gastric juice and mucosa by next generation sequencing method. J. Cancer Prev..

[84] Nardone G, Compare D, Rocco A (2017). A microbiota-centric view of diseases of the upper gastrointestinal tract. Lancet Gastroenterol. Hepatol..

[85] Tsuda A (2015). Influence of proton-pump inhibitors on the luminal microbiota in the gastrointestinal tract. Clin. Trans. Gastroenterol..

[86] Alarcón T, Llorca L, Perez-Perez G (2017). Impact of the Microbiota and gastric disease development by *Helicobacter pylori*. Curr. Topics Microbiol. Immunol..

[87] Yu G (2017). Molecular characterisation of the human stomach microbiota in gastric cancer patients. Front. Cell. Infect. Microbiol..

[88] Kim J (2015). An appropriate cutoff value for determining the colonization of *Helicobacter pylori* by the Pyrosequencing Method: Comparison with conventional methods. Helicobacter.

[89] Liu X (2019). Alterations of gastric mucosal microbiota across different stomach microhabitats in a cohort of 276 patients with gastric cancer. EbioMedicine.

