# Supplementary material for: Differential cytokine expression in gastric tissues highlights helicobacter pylori’s role in gastritis
Source: Sci Rep. 2024 Apr 1;14:7683. doi: 10.1038/s41598-024-58407-x (PMC10984929; doi:10.1038/s41598-024-58407-x)
Supplement: Supplementary file 1 — Supplementary Information. [file 41598_2024_58407_MOESM1_ESM.docx]

**Cytokine Expression Differences in Gastric Tissues with and without *Helicobacter pylori*-Associated Gastritis.**

Supplementary material

**Data S1. Materials and methods**

**Histology Studies.** The gastric mucosa was immediately (after dissection) perfused with 10% aqueous formaldehyde solution and then embedded in paraffin. Tissue sections of 5 µm were prepared using a microtome and stained with haematoxylin and eosin (H&E). The stained sections were analysed using a microscope slide scanner.

**Figure S1. Hematoxylin-Eosin Staining of Gastric Mucosa Samples from Gastric Biopsy.** This figure showcases the histological differences in gastric mucosa samples obtained from patients, divided into three groups for clarity. The normal group (N-group1, N-group2) displays gastric mucosa without pathological alterations. The normal gastritis group (G-group1, G-group2) and the *H. pylori*-infected gastritis group (GH-group1, GH-group2) exhibit various stages and characteristics of Gastritis. Key histological features highlighted with black arrows include eosinophil infiltration (EI), two forms of intestinal metaplasia (IM1: with glandular cavity; IM2: without glandular cavity of the pyloric gland), atrophy of the pyloric gland (APG), lymphocyte infiltration (LI), and the presence of *H. pylori* (HP) in GH-group samples. The scale bars provide a comparison of tissue architecture and inflammatory cell infiltration across different stages, with 100 µm scale bars for the N-group1, G-group1, and GH-group1 indicating a broader view, and 20 µm scale bars for N-group2, G-group2, and GH-group2 offering detailed observations. This visualisation aids in understanding the progression of histological changes from normal mucosa to non-infected and H. pylori-infected Gastritis, highlighting the inflammatory processes and structural changes across the groups.

**OLGA (operative link on gastritis assessment)**: OLGA ^2^ can be assessed through endoscopic examination and pathological analysis. The evaluation criteria include:

**Stage 0**: When the overall score for atrophy is 0 in both the mucosecreting and the oxyntic compartments (which means that none of the five standard biopsy samples show signs of atrophy), the OLGA stage is 0. The score for inflammatory lesions is independent of said stage, except in cases considered “indefinite for atrophy” because a florid inflammatory infiltrate may prevent the proper assessment of appropriate gland loss. All the Visual Analogue Scales (VAS) provided have been cleansed of any inflammatory component (to avoid confounding the issue), and no grading of the inflammatory lesions is mentioned. The VAS refer to non-atrophic (normal) mucosa and is given as a standard reference for comparisons with the “pathological” VAS.

**Stage I**: Stage I Gastritis is the lowest “atrophic” stage. In most cases (especially in *H. pylori*-infected patients), atrophic lesions are only detected in some biopsy samples. *H. pylori* status (positive versus negative) must be reported explicitly and is essential to the OLGA format. *H. pylori* may be difficult (or even impossible) to identify histologically at either antral or corpus level (particularly in patients on proton pump inhibitors [PPI]), in which case coexisting inflammatory lesions (polymorphs and lymphoid infiltrate) may suggest the bacterium’s presence even if it is not confirmed histologically. A comment on the suspected aetiology (“suspicious for H. pylori infection”) should be added in such cases (whatever their stage).

**Stage II**: This may result from a combination of different scores and locations of atrophic transformation. Atrophy can affect mucosecreting and oxyntic mucosa, but the atrophic lesions are often detected in the biopsy samples obtained from the mucosecreting area. *H. pylori* status (positive versus negative) has to be reported (see above). Based on preliminary experience with OLGA staging, stage II is the most represented in the low GC risk epidemiological setting.

**Stage III**: Stage III gastritis results from at least moderate atrophy at mucosecreting or oxyntic level. Atrophy is most frequently identified in the incisura angularis sample, and the most prevalent histological subtype of atrophic transformation is the metaplastic variant. Any presence of *H. pylori* needs to be reported (see above). When stage III is found in patients with no atrophy (score 0) in the biopsy samples of mucosecreting mucosa, the aetiological hypothesis of autoimmune (corpus-restricted) atrophic Gastritis should be considered. In most populations at low risk of GC, stage III is rarely encountered and may coexist with NiN or even more advanced (invasive) neoplastic disease.

**Stage IV**: Stage IV gastritis results in atrophy involving both antral and oxyntic mucosa, a situation corresponding to the panatrophic gastritis phenotype. In patients with *H. pylori* infection, extensive metaplastic transformation can interfere with the histological detection of the bacterium. This stage is rarely seen in areas with a low incidence of GC. Preliminary data show a strong association between OLGA stages III–IV and GC, so endoscopic surveillance programmes should focus on stage III–IV patients.

**Operative link on gastric intestinal metaplasia (OLGIM) assessment**: OLGIM ^3^ is an evaluation standard for evaluating the degree of gastric mucosal intestinal metaplasia. Gastric mucosal intestinal metaplasia resembles small intestinal epithelium and is characterised by well-developed goblet cells interspersed between columnar absorptive cells with a characteristic brush border. In contrast, the incomplete type has goblet cells of variable size and intervening columnar mucin-secreting cells without a brush border ^4^. Table S1 presents histological data for Gastritis and stages of OLGA and OLGIM across three groups: N-group, G-group, and GH-group. It categorises Gastritis into well-differentiated, moderately differentiated, and poorly differentiated, with corresponding counts for G and GH groups (N-group is not applicable here). For OLGA and OLGIM stages (ranging from 0 to IV), the table lists the number of cases in each stage for all three groups.

**Table S1: Histological grading of samples based on microscopic observation and criteria for OLGA and OLGIM stage**

|  | **N-group** | **G-group** | **GH-group** |
| --- | --- | --- | --- |
| **Gastritis:** |  |  |  |
| Well-differentiated | NA | 2 | 6 |
| Moderately differentiated | NA | 4 | 2 |
| Poorly differentiated | NA | 2 | 0 |
| **OLGA stage:** |  |  |  |
| 0 | 8 | 0 | 0 |
| Ⅰ | 0 | 3 | 2 |
| Ⅱ | 0 | 3 | 2 |
| Ⅲ | 0 | 2 | 4 |
| Ⅳ | 0 | 0 | 0 |
| **OLGIM stage:** |  |  |  |
| 0 | 8 | 4 | 0 |
| Ⅰ | 0 | 2 | 2 |
| Ⅱ | 0 | 2 | 2 |
| Ⅲ | 0 | 0 | 4 |
| Ⅳ | 0 | 0 | 0 |

**Endoscopy studies.** The Kyoto Classification of Gastritis, categorising *H. pylori* infection into three phases (non-gastritis, active Gastritis, and inactive Gastritis), was proposed to assess better the status of *H. pylori* infection and gastric cancer (GC) risk by endoscopy ^5^. In a healthy stomach, an easily detectable feature, non-gastritis, was the regular arrangement of collecting venules (RAC), featured as tiny red spots on the mucosal surface ^6,7^. However, after being infected with *H. pylori*, the stomach was characterised as irregular arrangement or absence of the so-called collecting venules ^8^. Atrophic Gastritis (AG) after infection by *H. pylori* presents with diffuse redness, spotty redness, mucosal oedema, and enlarged folds. This phenomenon can decrease and disappear after *H. pylori* eradication ^9,10^. After a period of *H. pylori* infection, Atrophic gastritis (AG) turns into inactive Gastritis upon eradication therapy or spontaneously disappears because of advanced atrophy, featuring map-like redness and flat or depressed erythematous tumours, which is the characteristic change of AG after *H. pylori* eradication, i.e., nonatrophied areas dissipate the inflammation. The atrophied areas are relatively red compared to the nonatrophied areas. Another characteristic was mottled patchy erythema (MPE) after *H. pylori* infection, showing many flat/slightly depressed erythematous lesions detected by white light endoscopy and highly predicting the impact of *H. pylori* eradication.

**Figure S2. Representative endoscopic features of different parts of gastrointestinal anatomy in different patient groups.**

Table S2 provides a comprehensive overview of endoscopic observations of various anatomical parts of the gastrointestinal system. The table categorises these observations across three groups, detailing the endoscopic features of each anatomical part, including the gastric fundus, body, antrum, angle, duodenum, cardia, oesophagus, and pylorus. Each section assesses aspects such as mucosa condition, mucus quantity, peristaltic movement, and the presence of ulcers.

**Table S2. Comparative endoscopic observations in gastrointestinal anatomy across different patient groups**

| **Anatomical part** | **Feature** | **N-Group** | **G-Group** | **GH-Group** |
| --- | --- | --- | --- | --- |
| Gastric fundus | Mucosa | Smooth | Smooth | Smooth |
|  | Mucus lake | Clear | Clear | Clear |
|  | Mucus amount | Medium | Medium | Medium |
| Gastric body | Mucosa | Smooth | Smooth | Smooth |
|  | Greater curvature of the mucosa | Wrinkled & Regular | Wrinkled & Regular | Wrinkled & Regular |
| Gastric antrum | Mucosa | Patchy hyperemic | Scattered in spots and patches of hyperemia, edema and erosion | Hyperemic and edematous, alternating with red and white blood vessels, |
|  | Peristaltic movement | Acceptable | Acceptable | Acceptable |
|  | Ulcer | No | No | A 0.3 cm ulcer was seen on the greater curvature, and the surface was covered with a white mass. |
| Gastric angle | Mucosa | Smooth and regular in shape | Smooth through blood vessels, and the shape is regular | The angle of the stomach was arched, and a 0.3 × 0.4 cm ulcer was seen in the middle of the stomach. The ulcer was covered with yellow-white moss and blood crust, and the surrounding mucosa was congested and swollen. |
| Duodenum | Mucosa | No abnormalities in the bulb and descending parts | No abnormalities in the duodenal bulb and descending parts | Large deep ulcers in the posterior wall of the bulb, with white fur and blood curst on the surface, and no abnormality in the descending mucosa |
| Cardia |  | Smooth, Open, and close | Smooth, Open, and close | Smooth, Open, and close |
| Oesophagus | Mucosa | Normal | Normal | Normal |
|  | Mucosal vascular texture | Regular | Regular | Regular |
|  | Dentate line | Regular | Regular | Regular |
| Pylorus |  | Round, Open and Close | Round, Open and Close | Round with normal switch function |

**References**

1.	Rugge, M. *et al.* OLGA Gastritis Staging for the Prediction of Gastric Cancer Risk: A Long-term Follow-up Study of 7436 Patients. *Am. J. Gastroenterol.* **113**, 1621–1628 (2018).

2.	Rugge, M. *et al.* OLGA staging for Gastritis: A tutorial. *Dig. Liver Dis.* **40**, 650–658 (2008).

3.	Capelle, L. G. *et al.* The staging of Gastritis with the OLGA system by using intestinal metaplasia as an accurate alternative for atrophic Gastritis. *Gastrointest. Endosc.* **71**, 1150–1158 (2010).

4.	Isajevs, S. *et al.* High-risk individuals for gastric cancer would be missed for surveillance without subtyping of intestinal metaplasia. *Virchows Arch.* **479**, 679–686 (2021).

5.	Tytgat, G. N. J. The Sydney System: Endoscopic division. Endoscopic appearances in gastritis/duodenitis. *J. Gastroenterol. Hepatol.* **6**, 223–234 (1991).

6.	Ebigbo, A., Marienhagen, J. & Messmann, H. Regular arrangement of collecting venules and the Kimura‐Takemoto classification for the endoscopic diagnosis of *Helicobacter pylori* infection: Evaluation in a Western setting. *Dig. Endosc.* **33**, 587–591 (2021).

7.	Zhao, J. *et al.* Accuracy of Endoscopic Diagnosis of *Helicobacter pylori* Based on the Kyoto Classification of Gastritis: A Multicenter Study. *Front. Oncol.* **10**, 1–9 (2020).

8.	Cho, J.-H. *et al.* Close observation of gastric mucosal pattern by standard endoscopy can predict *Helicobacter pylor*i infection status. *J. Gastroenterol. Hepatol.* **28**, 279–284 (2013).

9.	Nomura, S. *et al.* Endoscopic diagnosis of gastric mucosal activity and inflammation. *Dig. Endosc.* **25**, 136–146 (2013).

10.	Kato, M. *et al.* Changes in endoscopic findings of Gastritis after cure of *H. pylori* infection: Multicenter prospective trial. *Dig. Endosc.* **25**, 264–273 (2013).
